# Methylene blue administration in patients with refractory distributive shock – a retrospective study

**DOI:** 10.1038/s41598-020-58828-4

**Published:** 2020-02-04

**Authors:** Michal Porizka, Petr Kopecky, Helena Dvorakova, Jan Kunstyr, Michal Lips, Pavel Michalek, Martin Balik

**Affiliations:** General University Hospital, Department of Anaesthesiology, Resuscitation and Intensive Medicine, First Faculty of Medicine, Charles University in Prague, Prague, Czech Republic

**Keywords:** Bacterial infection, Combination drug therapy, Outcomes research

## Abstract

Hemodynamic effectiveness of methylene blue (MB) was tested in patients with refractory distributive shock. A retrospective analysis of 20 critically-ill patients who developed refractory shock was performed. Patients were divided into two study groups as responders with positive hemodynamic response to MB administration (defined as 10% decrease of norepinephrine dose) and non-responders. Hemodynamic, outcome data and baseline tissue hypoxia-related parameters including ratio of central venous-to-arterial carbon dioxide tension to arterio-venous oxygen content (P(v-a)CO_2_/C(a-v)O_2_) were compared between the groups. There were 9 (45%) responders and 11 (55%) non-responders to single bolus of MB administration. Dose of MB did not differ between responders and non-responders (1.3 ± 0.5 vs. 1.3 ± 0.4 mg/kg respectively, P = 0.979). MB responders had lower baseline P(v-a) CO_2_/C(a-v)O_2_ (1.79 ± 0.73 vs. 3.24 ± 1.18, P = 0.007), higher pH (7.26 ± 0.11 vs. 7.16 ± 0.10, P = 0.037) and lower lactate levels at 12 hours post MB administration (3.4 ± 2.7 vs. 9.9 ± 2.2 mmol/L, P = 0.002) compared to non-responders. Methylene blue represents a non-adrenergic vasopressor with only limited effectiveness in patients with refractory distributive shock. Profound tissue hypoxia with high degree of anaerobic metabolism was associated with the loss of hemodynamic responsiveness to its administration.

## Introduction

A circulatory shock with hypotension and the need for high vasopressor support in critically ill patients represents a state with excessive mortality exceeding 50%^1^. Its further progression into refractory shock, defined as norepinephrine requirements above 0.5 µg/kg/min^2^, is associated with even higher mortality^3^. Generally, any type of shock is associated with systemic tissue hypoperfusion leading to the development of systemic inflammatory response syndrome (SIRS) and generalized vasodilation, thus always progressing to some degree of distributive shock. Cytokine-induced hyperproduction of nitrous oxide, activation of endothelial ATP-sensitive potassium channels, vasopressin deficiency, and relative corticosteroid insufficiency are the key pathophysiological factors involved in this process^2^.

Recently, new rescue methods have been introduced in the management of refractory shock including the use of low-dose corticosteroid supplementation and non-adrenergic vasopressors administration such as vasopressin, angiotensin II and methylene blue^4,5^. Clinical trials have reported variable and limited hemodynamic responsiveness to these agents with documented dismal outcome in non-responders^5,6^, and the true effect on mortality remains unknown^7^.

Methylene blue (MB) is a reduction agent traditionally used in the treatment of methemoglobinaemia, however, it has been increasingly used in the management of refractory distributive shock. Its mechanism of action is based on the inhibition of the nitric oxide-cyclic guanosine monophosphate pathway leading to the increased vasomotor tone in the arterioles. The evidence consisting of case reports and several small clinical trials suggests that MB can be successfully used in patients with vasoplegic syndrome after surgery with the use of cardio-pulmonary bypass (CPB), septic shock or lethal dihydropyridine intoxication^8^. Nevertheless, similarly to the other non-adrenergic vasopressors, there is a lack of data on the actual MB hemodynamic responsiveness in patients with already established refractory shock and the factors that may influence it.

In this retrospective study, we evaluated the hemodynamic effectiveness of MB in the treatment of refractory distributive shock and its impact on clinical outcomes in a mixed population of medical and surgical patients.

## Methods

### Ethical approval and informed consent

1. Ethical approval for this study (201/19 S-IV) was provided by the Ethical Committee of General University Hospital, Prague, Czech Republic (Chairperson Prof. J. Sedivy) on 21^st^ February 2019.

2. Data acquisition and analysis were carried out in accordance with our institution´s local guidelines and regulations.

3. Due to the retrospective design of the study and according to national legislation Ethical Committee decided that patients´ informed consent was not required.

### Patient population

We searched our institution´s intensive care registry for any use of methylene blue (methylthionium chloride) as a vasopressor agent in the whole history of recorded data. Subsequently, identified patients´ data were collected and reviewed from hospital records. Only patients with refractory distributive shock with norepinephrine dose of more than 0.5 µg/kg/min and measured cardiac index of more than 2.4 L/min/m^2^ were included in the study.

### Study design

Patients were divided into two study groups. The first group comprised of patients who responded to MB administration by lowering the norepinephrine dose by 10% within the first 2 hours after the intervention. The patients who did not respond to the treatment were in the second group. In the previous studies, mean arterial pressure (MAP) change was used to assess vasopressor responsiveness^5,6^. However, in our institution, there is a nurse-driven protocol of norepinephrine infusion aiming for the MAP of 70 mmHg, therefore we used norepinephrine dose change instead. The demographic and medical history data, outcome data and baseline tissue hypoxia-related biochemical parameters including arterial lactate, pH, base deficit (BD), central venous oxygen saturation (ScvO_2_), central venous-to-arterial carbon dioxide tension difference (P(v-a)CO_2_ gap), ratio of central venous to arterial carbon dioxide tension to arterio-venous oxygen content (P(v-a)CO_2_/C(a-v) O_2_) and ionized calcium concentration were compared between the groups. Also, post-intervention 12-hour courses of norepinephrine doses, mean arterial pressures (MAP), arterial lactate and ionized calcium concentrations were compared between the groups.

### Statistical analysis

Statistica v.12 software (StatSoft, USA) was used for statistical analysis. The Kolmogorov-Smirnov test was used to verify the normality of distribution of continuous variables. Differences between the groups were assessed using Student’s t-test for normally distributed variables, which are expressed as mean ± standard deviation. A chi-square test, followed by Fisher’s exact test as appropriate, was used for comparisons of categorical data, which are expressed as counts and frequencies. 30-day survival was compared using the Kaplan-Meier method and the Mantel-Cox test was used to test survival differences between the study groups. P values < 0.05 were considered statistically significant.

## Results

Twenty patients who received a single intravenous bolus of MB for the treatment of refractory distributive shock were identified throughout the period from February 2011 to January 2019. Nine patients (45%) responded positively to MB administration and 11 patients (55%) were non-responders with an insignificant hemodynamic response. In responders, 6 patients (66.7%) were in post-CPB SIRS shock, 2 (22.2%) patients had septic shock and 1 patient (11.1%) suffered dihydropyridine intoxication induced distributive shock. In non-responders, there were only patients with post-CPB SIRS shock. Two patients (22.2%) in the MB responders group were on veno-arterial extracorporeal membrane oxygenation (ECMO). In these patients, MB was administered due to severe hypotension despite maximal ECMO blood flow rates of 6 L/min. Hemodynamic monitoring included the use of Swan-Ganz catheter in 12 (60%), Vigileo FloTrac^TM^ monitoring (Edwards Lifesciences) in 4 (20%) and transthoracic/transesophageal echocardiography in 4 patients (20%).

There was no significant difference in demographic or medical history data between the study groups (Table 1). All patients were intubated and mechanically ventilated, the degree of organ dysfunction assessed by SOFA and APACHE II score at the onset of refractory shock also did not differ between the study groups (Table 2). The dose of MB (1.3 ± 0.5 vs. 1.3 ± 0.4 mg/kg respectively, P = 0.979) and time from the onset of a refractory shock to intervention (12.7 ± 2.8 vs. 10.6 ± 6.8 hours respectively, P = 0.553) did not differ between responders and non-responders. There was not a significant difference in the use of other non-adrenergic vasopressors. A continuous infusion of vasopressin in the dose 4 IU/hour was administered in all responders and in 10 patients (92.9%) in the non-responder group (P = 1.00). Low-dose corticosteroid supplementation (intravenous boluses of hydrocortisone in the total daily dose of 2 mg/kg) was used in 4 (44.4%) responders and 3 (27.3%) non-responders (P = 0.642).

**Table 1:** Patient´s characteristics.

	Responders, n = 9	Non-responders, n = 11	P-value
Age (years)	57.9 ± 19.3	70.3 ± 8.1	0.068
Gender - males	5 (55.6%)	6 (54.5%)	0.091
BMI (kg/m^2^)	26.5 ± 5	26.3 ± 3.2	0.947
Hypertension	2 (22.2%)	8 (72.7%)	0.070
Diabetes mellitus	0	3 (27.3%)	0.218
COPD	2 (22.2%)	3 (27.3%)	1.000
Renal insufficiency	2 (22.2%)	4 (36.4%)	0.642
Peripheral vascular disease	2 (22.2%)	2 (18.2%)	1.000
CAD	1 (11.1%)	5 (45.5%)	0.157
LV EF (%)	54.3 ± 14.2	58.1 ± 11.8	0.526
NYHA I.	3 (33.3%)	0	0.074
II.	0	1 (9.1%)	
III.	6 (66.6%)	7 (63.6%)	
IV.	0	3 (27.3%)	

BMI, body mass index; COPD, chronic obstructive pulmonary disease; CAD, coronary artery disease; LV EF, left ventricular ejection fraction; NYHA, New York Heart Association heart failure classification.

**Table 2:** Patient´s baseline biochemical parameters and outcome data.

	Responders, n = 9	Non-responders, n = 11	P-value
Methylene blue dose (mg/kg)	1.3 ± 0.5	1.3 ± 0.4	0.979
Low dose corticosteroids	4 (44.4%)	3 (27.3%)	0.642
Vasopressin	9 (100%)	10 (90.9%)	1.000
Inotropes			
- dobutamine	2 (22.2%)	3 (27.3%)	0.795
- milrinone	0	1 (9.1%)	0.918
- levosimendan	0	1 (9.1%)	0.918
Baseline biochemical parameters			
- arterial lactate (mmol/L)	8.6 ± 5.6	11.4 ± 7.7	0.386
- arterial pH	7.26 ± 0.11	7.16 ± 0.10	0.037*
- base deficit (mmol/L)	5.5 ± 5.1	12.9 ± 3.2	0.001*
- ScvO_2_ (%)	68.6 ± 11.8	67.8 ± 13	0.821
- P(v-a)CO_2_ gap (mmHg)	7.84 ± 2.2	7.62 ± 2.42	0.842
- P(v-a)CO_2_/C(a-v)O_2_ (mmHg/mL)	1.79 ± 0.73	3.24 ± 1.18	0.007*
- ionized calcium (mmol/L)	0.90 ± 0.05	0.94 ± 0.13	0.517
SOFA score	14.2 ± 3.0	14.9 ± 1.8	0.556
APACHE II score	31.3 ± 4.7	32.1 ± 3.9	0.703
Mortality within 24 hours	0	5 (45.5%)	0.038*
ICU mortality	4 (44.4%)	11 (100%)	0.008*
30-day mortality	4 (44.4%)	11 (100%)	0.008*
ICU length of stay (days)	12.6 ± 11.9	2.5 ± 3.3	0.015*
Duration of mechanical ventilation (hours)	135 ± 111	51 ± 64	0.047*
ARDS	1 (11.1%)	2 (18.2%)	1.000
AKI	8 (88.9%)	9 (81.8%)	1.000
CRRT	2 (22.2%)	2 (18.2%)	1.000
VA-ECMO	2 (22.2%)	0	0.189

ScvO_2_, central venous oxygen saturation; P(v-a) CO_2_ gap, venous-to-arterial carbon dioxide tension difference; P(v-a)CO_2_/C(a-v)O_2,_ ratio of venous-to-arterial carbon dioxide tension to arterio-venous oxygen content; SOFA, Sequential organ failure assessment; APACHE II, Acute physiology and chronic health assessment; ICU, intensive care unit; ARDS, Adult respiratory distress syndrome; AKI, acute kidney injury; CRRT, continuous renal replacement therapy; VA-ECMO, veno-arterial extracorporeal membrane oxygenation.

*P < 0.05.

**Figure 1:**
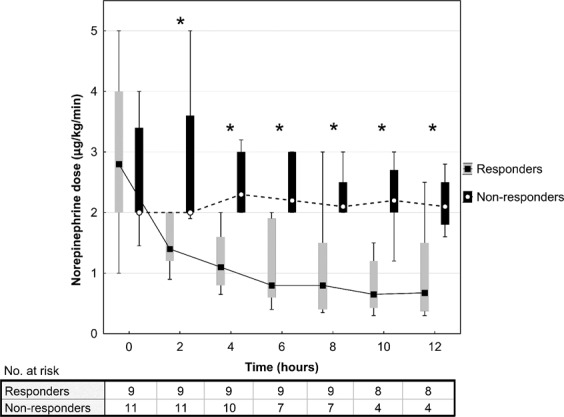
Norepinephrine requirements after methylene blue administration. Data are presented as box plot diagrams. The box represents the range of values (25th–75th percentile) with the horizontal line indicating the median, the whiskers depict adjacent values. *P < 0.05.

**Figure 2:**
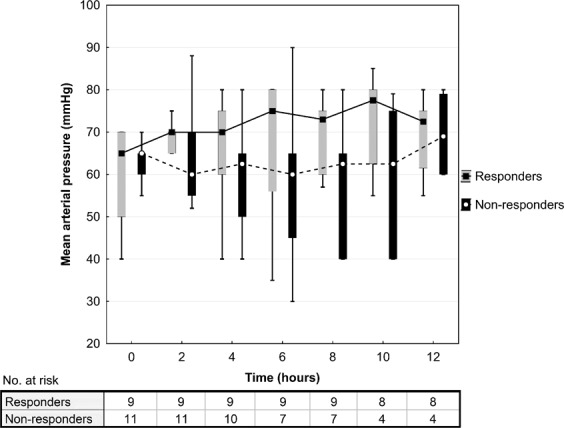
Mean arterial pressure after methylene blue administration. Data are presented as box plot diagrams. The box represents the range of values (25th–75th percentile) with the horizontal line indicating the median, the whiskers depict adjacent values.

Baseline norepinephrine doses did not differ between the groups, however after MB administration responders had significantly lower requirements in comparison to non-responders during the following 12 hours (Fig. 1). Mean arterial pressures did not differ between the study groups in the monitored 12-hour period (Fig. 2).

**Figure 3:**
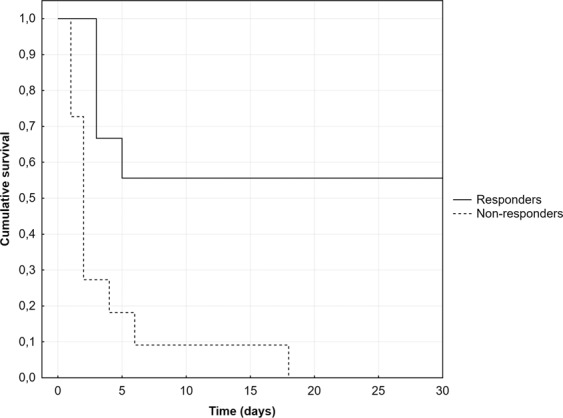
30-day survival: comparison between the methylene blue responders and non-responders.

There was no significant difference in the outcome data between the study groups (Table 2), except for the lower mortality within 24 hours from administration in the responders (0% vs. 45.5% in non-responders, P = 0.038) and lower ICU/30-day mortalities in the responders (both 44.4% vs. 100% in non-responders, P = 0.008). This was confirmed by Kaplan-Meier analysis that also showed a significant difference in 30-day survival between the study groups (P < 0.001) (Fig. 3). Due to this excessive 24-hour mortality in MB non-responders, responders had also significantly longer ICU stay (12.6 ± 11.9 vs. 2.5 ± 3.3 days respectively, P = 0.015) and duration of mechanical ventilation (135 ± 111 vs. 51 ± 64 hours respectively, P = 0.047) compared to non-responders.

**Figure 4:**
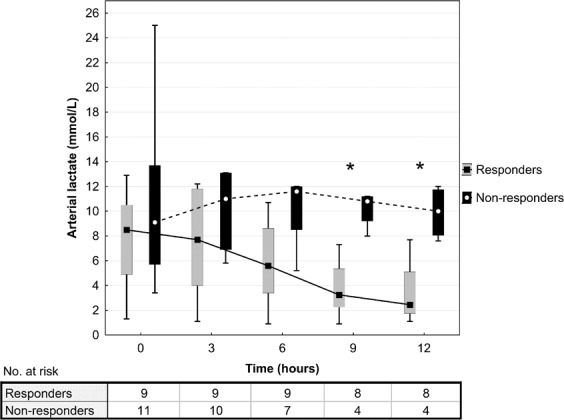
Arterial lactate concentrations after methylene blue administration. Data are presented as box plot diagrams. The box represents the range of values (25th–75th percentile) with the horizontal line indicating the median, the whiskers depict adjacent values. *P < 0.05.

**Figure 5:**
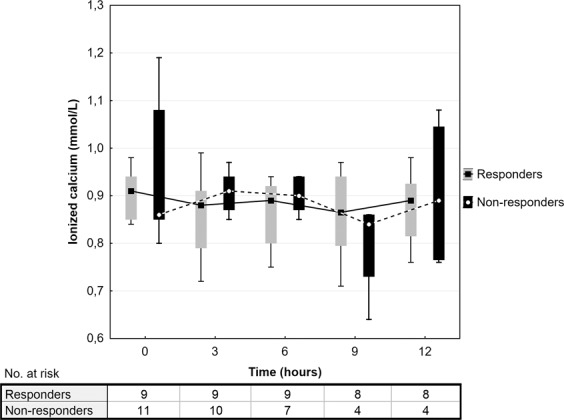
Ionized calcium concentrations after methylene blue administration. Data are presented as box plot diagrams. The box represents the range of values (25th–75th percentile) with the horizontal line indicating the median, the whiskers depict adjacent values.

In biochemical data analysis, there was a significantly higher baseline pH (7.26 ± 0.11 vs. 7.16 ± 0.10 respectively, P = 0.037) and lower BD (5.5 ± 5.1 vs. 12.9 ± 3.2 respectively, P = 0.001) in responders compared to non-responders (Table 2). Also, responders had lower P(v-a)CO_2_/C(a-v)O_2_ compared to non-responders (1.79 ± 0.73 vs. 3.24 ± 1.18 respectively, P = 0.007). Arterial lactate levels differed significantly between the responders and non-responders at 9 hours (4.2 ± 3.3 vs. 10.2 ± 1.5 mmol/L respectively, P = 0.007) and at 12 hours (3.4 ± 2.7 vs. 9.9 ± 2.2 mmol/L respectively, P = 0.002) after MB administration (Fig. 4). Ionized calcium concentrations did not differ between the study groups at any time point during the post-intervention 12-hour period (Fig. 5).

## Discussion

Our study showed limited hemodynamic responsiveness to MB administration of 45% in patients with refractory distributive shock. Those who responded had significantly improved survival compared to those with no hemodynamic response. Also, non-responders were in a more profound state of tissue hypoxia documented by significant metabolic acidosis and higher P(v-a)CO_2_/C(a-v)O_2_ compared to responders.

Methylene blue has been increasingly used as a non-adrenergic vasopressor in refractory shock management in various clinical scenarios including post-CPB, septic or dihydropyridine induced refractory shock. Many experimental studies reported beneficial hemodynamic effect, however clinical evidence is based only on case reports, small observational studies, and a few controlled trials^8^. The effect on patients´ outcome and mortality remains uncertain due to the study small sample sizes, which was also confirmed by the recent meta-analysis^4^. The majority of the above-mentioned studies compared MB administration to placebo or no treatment. Only one study reported responsiveness rate of 55% in an established distributive shock^9^, which is similar to our data. The lack of MB responsiveness could be caused by several factors including MB dosing regimen, a time delay between the onset of refractory shock and MB administration and finally by the actual severity of the patient´s clinical condition at the time of intervention.

Despite multiple performed studies, the adequate dosing regimen of MB remains unclear. The currently recommended dosing of 1–2 mg/kg used in experimental and clinical studies was originally based on the doses used for methemoglobinaemia treatment^8^. Also, various dosing regimens including single bolus, repeated boluses or bolus followed by infusion have been reported. In our study, only single boluses of MB were used in all patients and the doses did not differ significantly between responders and non-responders (Table 2). Therefore, we speculate that there should be other factors determining the final hemodynamic responsiveness to MB treatment.

Another parameter that may influence the MB responsiveness could be a time delay between the onset of refractory shock and MB administration. A time factor represents a key element in the concept of septic shock management as early interventions improve patients´ outcome^10^. This is supported by the recent study, in which vasoplegic cardiac surgical patients with early peroperative MB administration had improved outcome including mortality compared to the patients with late postoperative intervention^11^. This is in contrast with our study, in which the time from the onset of refractory shock (defined as norepinephrine dose more than 0.5 ug/kg/min) did not differ between the study groups (12.7 ± 2.8 hours in responders vs. 10.6 ± 6.8 hours in non-responders, P = NS). Thus, the time factor alone had no impact on the final MB responsiveness and the patient´s outcome.

Furthermore, global tissue hypoxia in shock states is the major trigger for the development of multiple organ dysfunction syndrome^12^. The commonly used indicators of tissue hypoxia including arterial lactate and central venous oxygen saturation may be unreliable in specific clinical situations. For instance, postoperative hyperlactatemia in cardiac surgery may not reflect only the presence of tissue hypoxia but can also represent a “wash-out” lactate after prolonged CPB, myocardial ischemia-reperfusion injury, or be the result of uncorrected hyperglycemia^13^. Therefore, novel parameters of tissue hypoxia including P(v-a)CO_2_ gap and P(v-a)CO_2_/C(a-v)O_2_ have been increasingly used in clinical practice. P(v-a)CO_2_ gap reflects flow-dependent CO_2_ removal from the microcirculation and represents a marker of global tissue hypoperfusion^14^. On the contrary, P(v-a)CO_2_/C(a-v)O_2_ is a surrogate of the respiratory quotient and is considered to be a marker of global tissue hypoxia and anaerobic metabolism^15^. In our study, MB non-responders had significantly increased.

P(v-a)CO_2_/C(a-v)O_2_ compared to responders (3.24 ± 0.11 vs. 1.79 ± 0.73 mmHg/mL respectively, P = 0.007) suggesting a deeper state of tissue hypoxia with higher degree of anaerobic metabolism. In the previous studies, high P(v-a)CO_2_/C(a-v)O_2_ has been associated with poor lactate clearance^16^ and with the condition of oxygen supply-dependency^15^, which are both associated with poor clinical outcome. This is in agreement with our study, where MB non-responders had significantly higher ICU mortality (100% vs. 44.4% in responders, P = 0.008) and lack of lactate clearance (Fig. 4) compared to responders. Additionally, MB non-responders were in more profound metabolic acidosis compared to responders (Table 2). Acidosis itself potentiates vasopressor hypo-responsiveness in refractory shock by activating ATP-sensitive potassium channels in vascular myocytes. This disables an increase of intracellular calcium and leads to further vasodilation^17^.

Finally, it has been reported that a low concentration of ionized calcium is common in patients with septic shock and its severity correlates with patient mortality^18^. Another study suggested that the correction of hypocalcaemia in critically ill patients reduced the doses of inotropes and vasopressors by improving myocardial contractility and vascular tonus^19^. However, recent human studies did not confirm these findings^20^ and even higher mortality was reported when calcium was supplemented in an animal experiment^21^. In our study, ionized calcium levels did not differ between MB responders and non-responders, even though they were mildly lowered in both groups with median values between 0.8–0.9 mmol/L during 12-hour post-intervention interval (Fig. 5). Therefore, we also conclude that ionized calcium concentration did not have a significant effect on MB responsiveness.

Based on our data, we assume that the patient´s responsiveness to MB may depend on the degree of actual tissue hypoxia and anaerobic metabolism. The mortality benefit seen in MB responders may likely be related to the lesser degree of tissue hypoxia rather than to the sole effect of MB and the decrease of vasopressor requirements. Thus, MB responsiveness could only be an indicator of the patient´s better clinical condition with the absence of irreversible hypoxic damage in the tissues. Nevertheless, we suggest that MB should be used as a rescue treatment of refractory distributive shock in patients who do not respond to standard treatment of volume expansion and catecholamine support. We also propose that MB should be administered early in the course of shock state before the profound tissue ischemic changes develop and vasoplegic syndrome progresses to its refractory phase. Further randomized controlled trials are required to elucidate the role of severity of tissue hypoxia on MB responsiveness in refractory shock, adequate MB dosing, MB effectiveness in different types of distributive shock and finally its effect on overall mortality of critically ill patients.

Methylene blue has a favorable safety profile. Minor side effects represent shortness of breath, nausea, vomiting, and discoloration of body fluids. Serious inadvertent effects including acute hemolytic anaemia^22^, detrimental effect on pulmonary functions^23^ and potentially fatal serotonin syndrome^24^ were reported only when high doses of MB were used. Except for the blue discoloration of body skin and urine, we have not detected any of these complications, probably due to the fact that the MB dose in our study was relatively low (1.3 mg/kg in both groups) and all patients were intubated and mechanically ventilated.

Our study has some limitations. First, the major one represents its retrospective design and the small number of patients, especially in interpreting outcome and mortality data. Second, due to the variable methods of hemodynamic monitoring including three different modalities we do not have sufficient data to demonstrate the effect of MB administration on the cardiac index and systemic vascular resistance. Third, the majority of patients in both study groups were classified as post-CPB SIRS shock. It is generally accepted that different pathogenetic mechanisms are involved in different types of vasodilatory shock to a variable extent^25^. Besides the inhibition of the nitric oxide-cyclic guanosine monophosphate pathway, the most significant factors include proinflammatory cytokines-induced impairment of endothelium-dependent dilatation, inhibition of vasodilator antiplatelet prostanoid^26^, activation of ATP-sensitive potassium channels and vasopressin deficiency^25^. Therefore, the hemodynamic response to MB may be variable in different types of distributive shock, which our study did not fully cover. Additionally, a relationship to prostaglandin inhibition may also associate with potentiation of coagulation in septic shock with a negative impact on capillary perfusion and patient´s outcome^27^. Fourth, the doses of norepinephrine used were extremely high in both study groups often exceeding the dose of 2 µg/kg/min. The pharmacodynamic effect of norepinephrine is based on the existence of the threshold concentration of 1000 pg/mL with a subsequent linear increase in effect as a function of the logarithm of concentrations^17^. The maximum effect is reached at the doses of 100 to 1000 times the threshold concentration and differs from one patient to another^17^. Thus, such extreme norepinephrine doses may be ineffective in some patients and the positive effect of MB could be then partially confounded by just simply lowering ineffectively high doses of norepinephrine.

In conclusion, methylene blue represents a non-adrenergic vasopressor with limited effectiveness in patients with refractory distributive shock. The development of profound tissue hypoxia and a high degree of anaerobic metabolism was associated with the loss of hemodynamic responsiveness to its administration.

## Data Availability

The datasets used and analyzed during the current study are available from the corresponding author on reasonable request.
